# Automatic evaluation of body-related words among young women: an experimental study

**DOI:** 10.1186/1471-2458-10-308

**Published:** 2010-06-04

**Authors:** Kaaren J Watts, Jacquelyn Cranney

**Affiliations:** 1 Psychosocial Research Group, Prince of Wales Hospital, Randwick, NSW, 2031, Australia; 2 School of Psychology, University of New South Wales, Kensington, NSW, 2052, Australia

## Abstract

**Background:**

Previous research has demonstrated that exposure to images depicting the thin female ideal has negative effects on some females' levels of body dissatisfaction. Much of this research, however, has utilised relatively long stimulus exposure times; thereby focusing on effortful and conscious processing of body-related stimuli. Relatively little is known about the nature of females' affective responses to the textual components of body-related stimuli, especially when these stimuli are only briefly encountered. The primary aim of the current research was to determine whether young women automatically evaluate body-related words and whether these responses are associated with body image concerns, including self-reported levels of appearance schematicity, thin internalisation, body dissatisfaction, and dietary restraint.

**Methods:**

An affective priming task was used to investigate whether females automatically evaluate body-related words, and whether this is associated with self-reported body image concerns. In a within-participants experimental design, the valence congruence of the prime and target pairs was manipulated. Participants selected body words as primes in Experiment 1 (*N *= 27), while normatively selected body words were primes in Experiment 2 (*N *= 50). Each prime was presented briefly, followed by a target word which participants judged as "good" or "bad". The dependent variable was response latency to the target.

**Results:**

Automatic evaluation was evident: responding to congruent pairs was faster than responding to incongruent pairs. Body image concerns were unrelated to automaticity.

**Conclusions:**

The findings suggest that brief encounters with body words are likely to prompt automatic evaluation in all young women, and that this process proceeds unintentionally and efficiently, without conscious guidance.

## Background

Body dissatisfaction is a prominent concern among females across the lifespan [1]. A large literature has established that body dissatisfaction is a causal risk factor in the development of body image disturbance and eating disorders, both of which seriously compromise physical and psychological health, with eating disorders associated with a disturbingly high mortality rate [2]. Sociocultural models have linked the development of body image disturbance and eating disorders to exposure to media messages promoting the thin female ideal [3]. Exposure to images depicting the slender female ideal, relative to exposure to average-sized or neutral images, has small yet consistent negative effects on some females' levels of body dissatisfaction; specifically, females with existing body image concerns are most likely to experience adverse effects [4]. The majority of this research, however, has utilised long exposure times and has, therefore, focused on effortful and conscious responses to the stimuli. Less is known, however, about the emotional impact of brief exposure to the symbolic or textual component of thin idealised messages, including words denoting appearance-related concepts. Fleeting exposure to body-related language may trigger fast, unintentional responses that are largely outside the individual's control. It is important to establish whether exposure to body-related words has this type of impact for two reasons. First, a large volume of appearance-oriented concepts are incidentally encountered in daily life, particularly in advertising, and there is evidence that linguistic features influence how advertisements are processed and elaborated [5]. Second, unintentional maladaptive responses to symbolic representations of body weight and shape may promote or underlie conscious and intentional negative evaluation of one's own body, or other-directed negative appraisals, including fat prejudice or discrimination.

There has been an increased focus on cognitive processes underlying the impact of media exposure [6,7]. Traditionally, cognitive theories distinguished between automatic and controlled modes of information processing. In other words, a process was either automatic, requiring minimal attentional resources, or controlled, such that the process demands substantial attentional resources [8]. However, this view of information processing has been challenged by more inclusive theories, such as the conditional model of automaticity [9]. In the conditional model, the key feature of an automatic process is that it is autonomous. That is, once the process starts it proceeds to completion without conscious guidance [10]. This model allows for several types of automatic processing, which are defined by the conditions that are present when the process occurs. For example, a post-conscious automatic process involves awareness of the triggering stimulus. However, once the process is initiated, it proceeds unintentionally and efficiently to completion, without intention or effort on the part of the individual [10].

Evaluation is one of the most dominant and pervasive of human responses [11], and the process of evaluation is often fast, efficient, and automatic [12]. Evaluative responses influence both the way we perceive and interpret stimuli in our environment [13] and our behaviour [14-16]. Automatic evaluation refers to activation of a positive or negative affective response upon brief exposure to a stimulus. Encounters with appearance-related stimuli, including body-related words, are often fleeting and are, therefore, likely to involve fast automatic processing [see [17]]. Brief exposure to body-related words or language in the media may involve conscious awareness of the stimulus, whilst the individual's evaluation of the stimulus is likely to be activated rapidly, efficiently, and unintentionally, without conscious effort. Such post-conscious automatic processing is likely to occur frequently as individuals encounter and process appearance-related stimuli on a daily basis. Despite this likely frequent phenomenon, automatic evaluation of body-related words has received less research focus than other information processing constructs, such as selective attention. Understanding automatic affective reactions toward body-related concepts is important because these processes are likely to be used as the basis for conscious evaluative judgments and attitudes [18], including those directed towards one's own and others' body and appearance.

### Automatic Evaluation and the Affective Priming Task

Indirect tasks provide the best means of assessing automatic, unintentional cognitions and attitudes because the participant is unaware of the processes that are being assessed [19]. The affective priming task (APT) involves participants categorising a target word as being positive or negative, with each target word preceded by another word called a 'prime' [20]. The key premise underlying the APT is that the experimenter can estimate the attitude toward the prime by examining how the presence of the prime influences the speed with which participants categorise the target word as 'positive' or 'negative'. The APT provides a measure of the *unintentional *nature of the process because participants are asked to engage in a process (evaluation of the target word) that is unrelated to the process of interest (evaluation of the prime) [9]. Further, the APT provides a powerful indirect test of automatic processing of the priming stimulus because participants do not respond directly to the primes and exposure to the priming stimuli is brief [< 500 milliseconds; 21]. Typically, two key features of each prime and target pair are manipulated to provide a test of post-conscious automatic processing of the prime. First, the emotional match (congruence) of the prime and target is varied; half of the pairs have the same valence (positive prime and positive target, or negative prime and negative target), and the remaining pairs have different valence (negative prime and positive target, or vice versa). These comprise the "congruent" and "incongruent" conditions, respectively. Second, the delay between the onset of the prime and the target, called the stimulus onset asynchrony (SOA), is often varied; half of the trials have a short delay (less than 500 ms) and the other half have a long delay (e.g., 1000 ms). The participant is instructed to look at the prime and to decide as quickly as possible whether the following target word is "good" or "bad", by pressing one of two response keys.

It is assumed that primes automatically activate responses on the basis of their valence [22,23]. For example, whenever a positive target word is presented (e.g., holiday), participants need to give a positive response (e.g., respond 'good'). When the target word is preceded by a positive prime (e.g., the word 'slender'), the positive valence of the prime will activate the tendency to produce a positive response to the target (e.g., respond 'good'), thereby facilitating the selection of a positive response to the target. However, when a positive target word is preceded by a negative prime (e.g., the word 'fat'), the prime will evoke a tendency to produce a negative response and will in turn, slow down the selection of the correct (positive) response to the target [23]. Hence, if automatic response activation is present, responses to congruent prime and target pairs will be faster than responses to incongruent pairs.

The manipulation of the SOA controls the amount of processing time available to participants and provides a test of the *efficiency *of the processing of the prime. At the brief SOA, the processing advantage afforded to congruent trials is optimal because the prime has just been presented. It is assumed that the congruence effect will not necessarily be evident at the long delay because (a) when given extra processing time, participants engage in intentional responding that may serve to suppress the effects of response activation, or (b) response activation has decayed by the time the target word is presented [20]. Hence, the classic criterion for automatic evaluation is established when congruent pairings are responded to faster than incongruent pairings at the short SOA but not at the long SOA (as indexed by the interaction between SOA and valence congruence). This pattern of responding has been replicated in several experiments using a variety of non-body related primes [24]. However, Fazio [25] has argued that the first condition, the presence of the congruence effect at the short SOA (the parsimonious criterion), is all that is needed to demonstrate automatic evaluation. Automatic evaluation of words according to this criterion has been demonstrated [24,26].

The strength of the association between the attitude object (the prime) and its evaluative node in memory (called "associative strength") has been examined in previous research as a potential moderator of automatic evaluation [25]. Specifically, it is postulated that exposure to "weak" attitude objects is less likely to trigger automatic evaluation than exposure to "strong" attitude objects. Fazio et al. [20] operationalised associative strength as speed of responding in a prime selection task. That is, prior to the APT, each individual was required to judge a set of potential primes as "good" or "bad" as quickly as possible. For each person, the words with the fastest response latencies were classified as "strong" primes, and those with the slowest response latencies were classified as "weak" primes. In several experiments, participants were faster to respond to congruent prime and target pairs than to incongruent pairs, but only when the primes were strong [20]. It is argued that the associative strength of body-related words in an individual's memory network also varies, and that some body-related words will be more likely to automatically activate affective responses than others.

### Individual Differences and Automatic Evaluation

Previous research has established that individual differences in body image concerns moderate the impact of exposure to thin idealised images [e.g., [4,27]]. Similarly, the magnitude of automatic processing of body-related words is likely to be influenced by individual differences in body weight and shape concerns. Cognitive models of body image disturbance predict that females who possess detailed mental representations of their body image (called body image schemas) will demonstrate more automatic processing of body-related stimuli [e.g., [28,29]]. Appearance schematicity comprises the cognitive-behavioural component of body image disturbance and it refers to the degree to which an individual personally invests in his or her physical appearance [30,31]. Appearance schematicity influences females' responses to body-related stimuli, including thin-idealised images [32] and appearance-related words [33]. A separate, but equally important construct is thin internalisation, which refers to the extent to which an individual endorses societal standards of size and attractiveness, and engages in behaviours designed to attain these ideals [34]. High thin internalisers are vulnerable to immediate adverse effects of thin idealised media [35], and in a meta-analysis, thin internalisation was one of three sociocultural variables that were related to negative body evaluation following exposure to thin idealised images [36]. The attitudinal component of body image disturbance is comprised of dissatisfaction with one's appearance, body shape, size, or specific body sites [30]. Body dissatisfaction has been examined extensively in experimental studies as a moderator of responses to thin idealised images. Females who are dissatisfied with their body respond more negatively to thin-idealised images than females who are less dissatisfied [4], and it is feasible that the former are more likely to attend to and to evaluate appearance-related words because these are highly relevant to their current body shape or weight concerns. Dietary restraint comprises attitudes toward eating, as well as effortful and goal-directed behaviours that are designed to regulate body weight [37]. Restrained eaters, in a similar manner to individuals who are dissatisfied with their body, are likely to attend to and to evaluate appearance-related stimuli because it is highly relevant to their goal of suppressing body weight.

The theoretical framework of post-conscious automatic processing together with a review of the empirical data, which suggest that automatic evaluation is a robust and replicable phenomenon, informed the current research. Watts, Cranney and Gleitzman [38] demonstrated that females automatically evaluate visual images of varying body shapes; however body shape messages can also be delivered through the symbolic media of oral and visual language which can be equally powerful. The automatic evaluation of body-related words has not, however, been systematically tested. In the first experiment; the automatic evaluation of body-related words, the potential moderating influence of associative strength, and the association with individual differences in body-related concerns, was tested for the first time. Based upon the findings of the first experiment, the methodology was refined and the automatic evaluation of body-related words was further examined, together with the potential moderating influence of appearance schematicity, in Experiment 2.

### Experiment 1

The primary objective of Experiment 1 was to determine whether females automatically evaluate body-related words and to determine whether these responses are moderated by the associative strength of the primes. Consistent with automatic evaluation previously demonstrated for nonbody-related words [24,26], it was predicted that responses to congruent trials would be faster than responses to incongruent trials at the short SOA but not necessarily at the long delay. A test of prime strength as a moderator variable would indicate that these response patterns were obtained for strong primes but not for weak primes. The second objective was to test the relationship between automatic evaluation and individual differences in concerns about appearance, weight, shape, and dieting. Although previous studies with other implicit paradigms [e.g., [14,39]] have demonstrated some such effects, Watts et al. [38] found no individual differences when images were used as primes with the APT. In view of the influence of body image concerns on females' responses to thin idealised media established in previous research, it was predicted that both the cognitive and attitudinal components of body image concerns (appearance schematicity, thin internalisation, body dissatisfaction and dietary restraint) would correlate positively with automatic evaluation of body-related words. The methodology used to test these predictions was based on the classic work of Fazio et al. [20].

## Methods

### Participants and Design

Experiment 1 employed a (2 × 2 × 2) within-participants factorial design. The first factor was the delay between the onset of the prime and the target (SOA). In half of the trials the SOA was short (300 ms) and in the other half it was long (1000 ms). The second factor was prime strength (strong, weak). The valence congruence of the prime and target pairs was the third factor. Each prime and target pair had either the same valence (congruent) or different valence (incongruent). Half of the trials were congruent and half were incongruent. The dependent measure was the mean response latency (ms) to the target words. Twenty-eight female undergraduate psychology students at the University of New South Wales (UNSW) participated in this experiment in return for course credit. The data set for one participant was excluded because of a high error rate. The mean age of the final sample (*N *= 27) was 20.22 years (*SD *= 6.93 years) and the mean body mass index (BMI) was 20.48 (*SD *= 3.00).

Approval for Experiments 1 and 2 was obtained from the UNSW Human Research Ethics Committee.

### Materials

The experimental stimuli were presented on personal IBM compatible computers using Inquisit (version 2.0; Millisecond Software LLC., 2004).

*Prime selection task*. The stimuli consisted of 92 body-related nouns. The words were selected by the experimenter in order to reflect a range of body-related concepts including body parts (e.g., *hips, stomach, eyes*) and body shape and weight (e.g., *cellulite, thinness, obesity*).

*APT*. Eight positive and eight negative body-related words were extracted for each participant from the prime selection task and these comprised the primes in the APT. Consistent with the procedure used by Powell and Fazio [40] and Fazio et al. [20], half of the primes were classified as "strong" and half as "weak", based upon each individual's fastest and slowest response latencies, respectively. Hence, there were five categories of primes: strong-good, strong-bad, weak-good, and weak-bad, and a nonprime baseline (four primes per category). Each prime was followed by a target adjective which the participant was required to respond to. Ten of the target words were positive (e.g., *beautiful, magnificent) *and 10 were negative (e.g. *awful, miserable*). The target words were identical to those employed by Watts et al. [38]. The target words were matched for frequency of usage, number of syllables, and word length.

### Measures

*Appearance Schemas Inventory-Revised (ASI-R)*[31]. The ASI-R (20 items) is designed to assess self-schemas pertaining to the degree of personal investment in physical appearance. Responses are made on a 5-point scale ranging from 1 *(strongly disagree) *to 5 *(strongly agree)*. The composite ASI-R score is obtained by summing the scores for each item and taking the mean of the total (score range 1-5) with higher scores indicating a higher level of appearance schematicity. The scale produces reliable and valid scores in females aged 18 years and over [31,41,42]. In the present study good internal consistency was demonstrated (α = .87).

*Sociocultural Attitudes Towards Appearance Scale-3: Internalisation-General Subscale (SATAQ-3-I)*. The SATAQ-3-I (9 items) [43] measures the extent to which thin appearance ideals have been endorsed as personal standards that are desired or strived for. For example, "I would like my body to look like the people who are on TV". Participants indicate their level of agreement with each statement using a 5-point scale ranging from 1 *(definitely disagree) *to 5 *(definitely agree)*. Scores for each item are summed (score range 9-45), with higher scores indicating greater internalisation of the thin ideal. The scale has good psychometric properties and has been validated with adult females [43]. Internal consistency was excellent in the present study (α = .91).

*Restraint Scale (RS)*. The RS (11 items) [44] measures attitudinal and behavioural concerns about dieting and weight regulation. For example, "what is the maximum amount of weight that you have ever lost in 1 month?" The scale was modified for an Australian sample (units were changed from pounds to kg), and items 2, 3, 4, and 11 were formatted so that the participant could enter a specific weight, instead of using the standard forced choice format. Given the differences in the weight range choices provided in the original items, items 3 and 4 were scored using one point per 1 kg, and items 2 and 11 were scored using 1 point per 2 kg. The other items were scored in the standard way. Responses to the 11 scored items were then summed, with high scores representing a high level of dietary restraint [37]. The scale produces reliable and valid scores in college-aged females [37,45]. Internal consistency in the current experiment was good (α = .78).

*Eating Disorder Inventory: Body Dissatisfaction Subscale (EDI-BD)*. The EDI-BD (9 items) [46] is a measure of dissatisfaction with one's shape and with the size of specific body sites. For example, "I think that my stomach is too big". Participants indicate whether each statement applies to them (6 *always *to 1 *never)*. The inventory was scored as a continuous measure (score range 9-54), with a higher score indicating greater body dissatisfaction [47]. The scale demonstrates high internal consistency [48] and good validity in adult females [46]. Cronbach's alpha in the current experiment was excellent (α = .92).

### Procedure

The current experiment was run concurrently with another experiment (not reported here), and the order of participation was counterbalanced. The other experiment was identical to Experiment 1 with the exception that the primes were nonbody-related words. Because both studies tested post-conscious automatic processes, it was expected that the experiment completed first would impact minimally upon responses in the other, and this expectation was confirmed by data analyses. Prior to the experiment that was administered first, participants were told that the study was concerned with females' thoughts and feelings about words commonly used in advertising. The questionnaires were completed first, followed by the prime selection task and then the APT for each experiment.

*Prime selection task*. This task involved the computerised presentation of 92 body-related nouns. The participant had to judge as quickly as possible whether each word was "good" or "bad" by pressing the "z" or the "1" key on a standard keyboard (labelled "G" and "B", respectively). Each word remained on the screen until the participant gave a valid response. Ten practice trials were administered. The intertrial interval (ITI) was three seconds, consistent with Fazio et al.[20]. At the completion of the task, an Excel macro (version 11.0) extracted a set of 16 primes for each individual and inserted these as the priming stimuli into the software for the APT. The primes consisted of eight nouns with the fastest response latencies and eight nouns with the slowest response latencies. There were an equal number of words of positive valence and negative valence in each category.

*APT*. Each trial involved the presentation of a prime followed by a target adjective. Participants were instructed to look at the first word, commit it to memory, and then decide quickly and accurately whether the target word was "good" or "bad" by pressing the key labelled "G" or "B", respectively. Immediately following their evaluation of the target, they were to (silently) recall the prime. To ensure that participants attended to the primes, they were told that they would be tested on their memory for the word stimuli at the end of the experiment. Each prime appeared on the screen for 200 milliseconds. At the short SOA, the interval between the offset of the prime and the onset of the target word was 100 ms, giving an SOA of 300 ms (SOA 300). At the long SOA, the interval was 800 ms, giving an SOA of 1000 ms (SOA 1000). Following Fazio et al. [20], the ITI was four seconds.

The SOA conditions were presented in separate blocks (order counterbalanced). Five blocks of trials were presented in each SOA condition. Each block consisted of 20 trials, with each of the 20 primes (including the letter strings) and 20 target adjectives presented once. For each of the five categories of primes, half of the primes were followed by positive adjectives and half by negative adjectives. Across the five blocks, each target adjective was paired once with a prime from each of the five prime categories. Thus, there were 100 trials in each SOA condition, and 200 trials overall. To minimise fatigue, a 10 second break was included midway through each SOA block and participants were permitted to rest briefly between the two blocks of trials.

In both tasks, each word was presented in the centre of a computer screen in black uppercase letters in Arial font (0.8 mm high), in a 110 mm × 15 mm white box that, in turn, was set upon a light blue background. Key assignment for "good" and "bad" responses was counterbalanced across participants, and this assignment was consistent across tasks for each individual. A reminder of the response options "good" and "bad" appeared in the top left and right corners of the screen in black uppercase letters. Each target word remained upon the screen until a valid response was given. Upon completion of the experiment, participants were thanked for their participation and were fully debriefed.

### Data Reduction and Statistical Analyses

As is standard in this paradigm, incorrect responses were defined as valence judgments opposite to the actual valence of the target word (e.g., responding "good" to a negative target). These comprised 4.54% of the total trials after the practice trials were removed. These were coded as errors and were excluded from the data analyses. Prior to the calculation of the dependent variable, outlying response latencies were dealt with by a process called "winsorising" [49]. This involves replacing latencies that are more than two standard deviations (*SDs*) above (or below) the individual's mean (the criterion) with the value that is exactly two *SDs *above (or below) the mean. In the current experiment, these trials comprised 4.37% of the total trials. Consistent with other indirect paradigms, including the Implicit Association Test (IAT) [50], the index of automatic evaluation was conceptualised in the current study as the difference in response latencies between congruent and incongruent trials. The criterion was calculated by taking the difference between mean response latencies for congruent and incongruent trials at the short SOA (i.e., Mean_Short Incongruent _-- Mean_Short Congruent_). A larger positive difference indicated greater automaticity. The association between automatic evaluation and body image concerns was tested by correlating the scores for appearance schematicity, thin internalisation, body dissatisfaction, and dietary restraint with the index of automaticity. A significance level of alpha equals .05 was used for all analyses. The means of the dependent variables were compared by analysis of variance (ANOVA). We did not control for body mass index (BMI) because it was not correlated with any of the response latencies at either the short or the long SOA.

## Results and Discussion

### Sample Characteristics

Table 1 presents the mean sample characteristics. The means in the current experiment for BMI, appearance schematicity, thin internalisation, and body dissatisfaction were all within the range reported in previous research with college-aged females [31,51-53]. The median restraint score for college-aged females generally ranges from 15 to 16 [37]. The median in the current sample was somewhat higher (median = 18.5).

**Table 1:** Mean Sample Characteristics in Experiment 1 and Experiment 2

Experiment 1			Experiment 2			
(N = 27)			Aschematic (n = 23)		Schematic (n = 27)	
	*M*	*SD*	*M*	*SD*	*M*	*SD*
Age (years)	20.22	6.93	19.74	3.65	19.78	2.89
BMI	20.48	3.00	22.48	4.62	21.00	3.56
ASI-R	3.36	0.55	2.39^a^	0.28	4.45^b^	0.27
SATAQ-3-I	29.30	7.31	19.48^a^	7.82	34.89^b^	6.53
EDI-BD	33.11	10.73	29.09^a^	11.44	38.11^b^	10.12
RS	18.43	7.11	16.34^a^	6.42	24.21^b^	8.66

*Note*. BMI = Body Mass Index; ASI-R = Appearance Schemas Inventory-Revised; SATAQ-3-I = Sociocultural Attitudes Towards Appearance Scale-3, Internalisation General subscale; EDI-BD = Eating Disorder Inventory, Body Dissatisfaction subscale; RS = Restraint Scale. Means within a row with different superscripts are significantly different at *p *< .05.

### Order Effects

To examine potential order effects a 2 (Order of SOA) × (2) (SOA) × (2) (Prime Strength) × (2) (Congruence) ANOVA of mean response latencies was conducted. The main effect of SOA was qualified by a significant interaction between order of SOA and SOA condition, *F*(1, 25) = 9.37, *p <*.01, partial η^2 ^= 0.27. Group 1 (SOA 300 first), were slower to respond to short trials than to long trials, and Group 2 (SOA 1000 first) yielded the opposite pattern. This pattern is consistent with practice effects such that each group became more proficient at the task as they progressed through it. A one-way ANOVA, however, conducted on the absolute difference in latencies between SOA 300 and SOA 1000 trials confirmed that the size of the order effect did not differ significantly between Group 1 and Group 2, *F*(1, 25) = 2.29, *p *> .05. Therefore, subsequent analyses were collapsed across the order of SOA variable, and this is consistent with the procedure of Fazio et al. [20: Experiment 2].

### Automatic Evaluation

**Figure 1:**
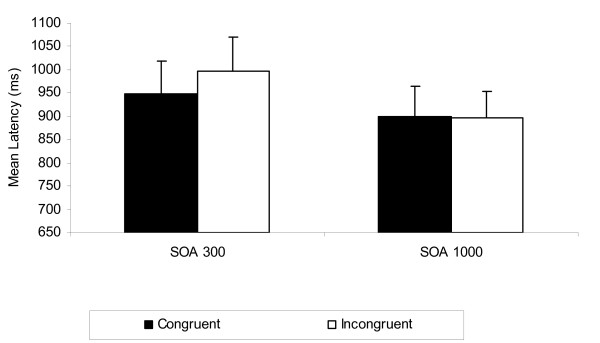
**Experiment 1 mean response latency (ms) as a function of SOA and valence congruence**.

Figure 1 suggests that consistent with expectations, response latencies were faster for congruent trials than for incongruent trials at the short SOA but not at the long SOA. The initial order analysis provided a test of the classic criterion. There was a significant interaction between SOA and congruence, *F*(1, 25) = 9.03, *p <*.01, *MSE *= 4,370.65, partial η^2 ^= .27, and this qualified a main effect of congruence, *F*(1, 25) = 6.50, *p <*.05, *MSE *= 4,749.55, partial η^2 ^= .21. To test the parsimonious criterion, a (2) (Prime Strength: strong, weak) × (2) (Congruence: congruent, incongruent) ANOVA was conducted on the SOA 300 latencies. Response latencies for congruent trials were significantly faster than response latencies for incongruent trials at the short delay, *F*(1, 26) = 17.88, *MSE *= 3,781.13, *p <*.001, partial η^2 ^= .41. There was no moderating effect of prime strength.

### Individual Differences and Automatic Evaluation

Pearson product moment correlations were conducted to test the relationship between body image concerns and automatic evaluation (see Table 2). Each of the individual difference variables intercorrelated to at least a moderate degree, but none correlated reliably with the criterion of automatic evaluation. Hence, contrary to expectation, automatic evaluation of body-related words was not associated with appearance schematicity, thin internalisation, body dissatisfaction, or dietary restraint.

**Table 2:** Pearson product moment correlations between the individual differences and the criterion of automatic evaluation (Mean_Short Incongruent _- Mean_Short Congruent_) in Experiment 1

		1	2	3	4	5
1.	SATAQ-3-I	1.00	.69**	.27	.22	.00
2.	ASI-R		1.00	.39*	.35	-.07
3.	EDI-BD			1.00	.82**	.25
4.	RS				1.00	.19
5.	Criterion Variable					1.00

*Note*. SATAQ-3-I = Sociocultural Attitudes Towards Appearance Scale-3, internalisation general subscale; ASIR = Appearance Schemas Inventory-Revised; EDI-BD = Eating Disorder Inventory, body dissatisfaction subscale; RS = Restraint Scale. *p < .05. **p < .01.

Automatic evaluation according to the classic criterion was obtained following brief exposure to body-related words. Two aspects of the priming task suggest that these responses involved post-conscious automatic processing. First, at the short delay, participants did not have time to implement an intentional, deliberate response [20,21]. Second, the predicted congruence effect occurred even though the participants were not asked to explicitly report their attitudes toward the primes during the priming task. The absence of affective priming at the long delay is consistent with models of memory retrieval which postulate that response activation is temporary and decays rapidly [e.g., [54,55]].

Contrary to expectation, the associative strength of the primes did not moderate automatic evaluation. This, however, is consistent with previous research in which body-related images were employed as primes [38]. Together, these findings for body-related stimuli highlight the ongoing debate in the affective priming literature [56,57] that challenges Fazio et al.'s [20] conclusion that the accessibility of an attitude from memory is determined by the strength of the associative link between the attitude object and its evaluation, as measured by prime evaluation response latencies. The present results suggest that a variety of body-related words are likely to activate automatic responses, irrespective of the strength of their association in memory.

A potential limitation of Experiment 1 was that the initial prime selection task may have temporarily primed object-evaluation associations which in turn, produced the affective priming effects rather than automatic activation. That is, the congruence effects may reflect temporary activation arising from participants having just evaluated the body concepts in the prime selection task [24]. The first objective of Experiment 2 was to establish whether automatic activation of attitudes toward body-related primes remained when a set of normatively- rather than individually- selected body-related primes was used. Importantly, if the priming effect remains, this will suggest that this automatic phenomenon is relatively unconditional and pervasive.

The hypothesis that individual differences in body image concerns would correlate with automatic evaluation was not supported. The sample size was small and the power to detect the hypothesised relationships was not optimal. Nevertheless, cognitive models of body image disturbance postulate that individuals with complex and highly integrated self-schemas containing thoughts, feelings, and beliefs about their body image, process appearance-related information differently to individuals who have less complex body image schemas [e.g., [28,29]]. Indeed, there is evidence that individuals who invest highly in their physical appearance ("schematics") selectively attend to body-related words, and also exhibit greater body dissatisfaction upon exposure to thin idealised media, than "aschematic" individuals [32,33]. Moreover, high levels of appearance schematicity have been shown to correlate with dysfunctional attitudes toward one's body image [31,42,58]. It is possible, therefore, that individual differences in automatic evaluation may emerge only in females who are excessively concerned with their physical appearance and who view it as a critical component of their self-identity.

The second objective of Experiment 2 was to test whether the predicted difference in automaticity would be apparent when individuals were selected on the basis of extreme low and high scores on the measure of appearance schematicity. Participants were screened and recruited on the basis of extreme scores falling at the upper and lower end of the Appearance Schemas Inventory-Revised, and their responses on the APT were compared. This is in keeping with the procedure adopted by Lavin and Cash [59] who classified individuals scoring in the upper tertile of the Appearance Schemas Inventory [60] as "schematic", and those scoring in the lower tertile as "aschematic". It was predicted that schematics would show greater automatic evaluation than aschematics. This would be demonstrated by (a) a three-way interaction between schematicity, SOA, and congruence, according to the classic criterion, or (b) by a simple interaction between schematicity and congruence at the short SOA, consistent with the parsimonious criterion.

### Experiment 2

## Methods

### Participants and Design

Participants were selected on the basis of their scores on the ASI-R [31], collected in an earlier mass screening conducted with first year psychology students at the University of New South Wales. The final screening sample consisted of 296 female undergraduates who completed and returned the ASI-R (*M *= 3.42, *SD *= 0.63). Individuals with a mean score more than one SD below the screening sample mean were classified as aschematic and those with a mean score more than one SD above the mean were classified as schematic. One standard deviation above or below the mean ASI-R score for the screening sample was used as the criterion for classification of participants as schematic or aschematic, rather than the stricter criterion of the upper and lower tertiles, because of the inherent difficulties with missing data due to participant attrition over subsequent testing sessions (not reported here). Fifty of these females were contacted via telephone and participated in the current study in return for course credit. Twenty-three were aschematic (mean composite score on the ASI-R '*M*_ASIR_' = 2.39, *SD *= 0.28) and 27 were schematic (*M*_ASIR _= 4.45, *SD *= 0.37), with a mean age of 19.76 years (*SD *= 3.22) and a mean BMI of 21.68 (*SD *= 4.10). A 2(Schematicity: Aschematic, Schematic) × (2) (SOA: Short 300 ms, Long 1000 ms) × (2) (Valence Congruence: Congruent, Incongruent) mixed design was employed. The dependent variable was the mean response latency (ms) to the target words.

### Materials

Twenty pairs of primes and targets comprised the stimuli in the computerised APT. The 20 body-related primes (10 positive and 10 negative) were selected normatively on the basis of evaluative judgments obtained from 16 female graduate psychology students who did not participate in the current experiment. These students judged whether 95 body-related words (presented via computer) were "good" or "bad" as quickly as possible. The stimuli included the 92 body-related words from Experiment 1 and three additional words *(overweight, petiteness, slimness)*. To ensure maximal agreement on the valence of the primes, the twenty words rated most consistently as either "good" or "bad" were selected as primes. The words in each of the prime categories "good" and "bad" did not differ significantly in terms of mean length or mean frequency. The majority of words in each category were body shape concepts (e.g., *bulge, cellulite, slimness) *rather than body parts (*hips*, *knees, legs)*. The target adjectives (10 positive and 10 negative) and the questionnaires were identical to those in Experiment 1.

### Procedure

Participants were told that the study was concerned with young women's emotional responses to health-related words and how these are influenced by individual differences in health-related beliefs and values. The APT was completed and participants were fully debriefed after completion of a further study (not reported here). The questionnaires were completed in a separate session one week later.

In relation to the APT, the presentation of the word stimuli, the prime memory instructions, the mode of response and response key assignment were identical to that used in Experiment 1 with two exceptions. An intertrial interval (ITI) of 2.5 seconds was used compared to the ITI of 4 seconds employed in Experiment 1. The shorter interval was used in order to minimise eye fatigue. This interval has been used successfully by Roefs et al.[26]. Second, to ensure that participants focused on the screen, a black fixation cross was introduced to signal the beginning of each trial. The cross appeared in the centre of the screen for 500 ms before each prime. A pilot study confirmed that participants found the fixation cross useful for focusing their attention. The prime immediately followed the offset of the fixation cross. Thereafter, the time course of each trial was identical to that of Experiment 1.

The way in which the prime and target stimuli were paired within each of the two blocks of trials was identical to the approach in Experiment 1 with two exceptions. First, fewer trials were required because there were only two categories of primes ("good" and "bad"), rather than five prime categories. Therefore, within each SOA condition, two (rather than five) blocks of trials were presented giving a total of four blocks of trials in this study. Each block consisted of 20 trials giving a grand total of 80 trials. To reduce the substantial "order of SOA" effects obtained in Experiment 1, the number of practice items administered prior to the experimental trials was increased to twenty.

### Data Reduction and Analyses

Incorrect responses comprised 3.88% of the total trials after the practice trials were removed. As in Experiment 1, these were coded as errors and were excluded from the data analyses. Outlying response latencies were winsorised prior to the calculation of the mean response latencies, also comprising 3.88% of the total trials. The means of the dependent variables were compared by ANOVA and multivariate analysis of variance (MANOVA).

## Results and Discussion

### Sample Characteristics

The mean participant characteristics are reported in Table 1, and are similar to those reported for Experiment 1. A MANOVA confirmed that there were no significant differences between the low (aschematic) and high (schematic) groups in age, *F*(1, 46) = .004, *p > .05*, or BMI *F*(1, 46) = 1.57, *p *> .05. There were significant differences for all other variables, as might be expected, particularly given the significant intercorrelations reported in Experiment 1. The group differences in Experiment 2 are consistent with the strong positive correlation that appearance schematicity demonstrates with other body image variables [e.g., thin internalisation, [31]. Cronbach's alpha for the measures of appearance schematicity, thin internalisation, dietary restraint, and body dissatisfaction in the present experiment was .97, .97, .74, and .93, respectively.

### Order Effects

To examine potential order effects a 2 (Order of SOA) × 2 (Schematicity) × (2) (SOA) × (2) (Congruence) ANOVA was conducted on mean response latencies. The main effect of order of SOA was not significant, *F*(1,46) = .070, *p > .05*, nor was the four-way interaction, *F*(1,46) = 2.03, *p > .05.*

### Automatic Evaluation

**Figure 2:**
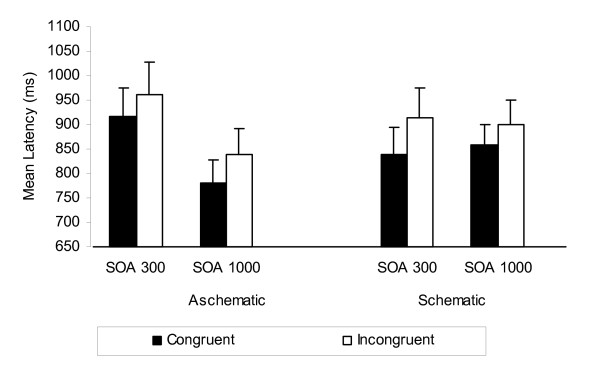
**Experiment 2 mean response latency (ms) as a function of appearance schematicity, SOA, and valence congruence**. Reproduced from *Current Psychiatry Reviews *2009;5:110-126 with the permission of Bentham Science Publishers Ltd and the authors Watts and Cranney (2009).

Figure 2 suggests that on average, response latencies were faster for congruent trials than for incongruent trials. The highly significant main effect of congruence confirmed this pattern, *F*(1, 46) = 18.93, *MSE *= 8,207.19, *p *< .001, partial η^*2 *^= 0.29. Contrary to classic criterion predictions, the interaction between SOA and valence congruence was not significant. Consistent with the parsimonious criterion, responses to congruent trials were significantly faster than responses to incongruent trials at the short SOA, *F*(1, 48) = 14.60, *MSE *= 6,401.77, *p *< .001, partial η^*2 *^= 0.23. There were no main or interaction effects for schematicity, with one exception. Aschematics responded more quickly to SOA 1000 trials than to SOA 300 trials, compared to schematics, *F*(1, 46) = 4.24, *p *= .05, partial η^*2 *^= 0.08, suggesting that schematics may have engaged in more elaborative, effortful processing of body-related words when they had the time to do so.

Consistent with the parsimonious criterion, responses to congruent trials were faster than responses to incongruent trials at the short SOA. Hence, automatic responding was obtained with normatively selected primes in the absence of the initial prime selection task. This confirms that automatic evaluation of body-related words is not conditional upon temporary activation produced by the prime selection task. Contrary to expectation, automaticity was not influenced by appearance schematicity; schematics and aschematics showed a similar processing advantage for congruent trials relative to incongruent trials at the short SOA. This is consistent with Experiment 1 in which appearance schematicity (as a continuous measure) was not significantly associated with automatic evaluation.

## General Discussion

The results of these experiments demonstrated that the written word can induce automatic evaluation of body shape and weight. Specifically, and consistent with expectations, responses to congruent prime and target pairs were faster than responses to incongruent pairs at the short SOA in both experiments. The presence of the congruence effect at the short SOA suggests that females' affective responses were automatically activated upon brief exposure to body-related words. Moreover, the indirect nature of the priming task highlights that participants' responses were autonomous, unintentional and automatic. That is, despite participants being instructed to evaluate the target words and not the primes, congruent trials were afforded a significant processing advantage relative to incongruent trials at the short SOA when participants did not have sufficient time to plan their responses [20,24]. The primes were presented briefly for 200 ms and participants were asked to retain each prime in memory. This encouraged participants to attend to the primes and to become consciously aware of the body-related words. However, at the short SOA the response activation that followed was rapid, unintentional and efficient. Hence, the pattern of responding at the short SOA is consistent with the post-conscious mode of information processing postulated in the conditional model of automaticity [10]. That is, the affective responses activated by the body-related primes proceeded rapidly and unintentionally, without conscious guidance or monitoring by participants.

The congruence effect persisted at the long SOA in Experiment 2, but not in Experiment 1. At the long delay, participants had sufficient time to implement controlled, goal-directed responses [25]. In Experiment 2, therefore, it appears that even though participants had the opportunity to change the nature of their responses at the long SOA, they did not do so. Why might this be so? The evaluation of thinness as positive and overweight as negative reflects Western society's idealisation of slenderness and denigration of fatness [61]. Indeed, motivation to control fat prejudice is often low [62] and explicit anti-fat attitudes, prejudice and discrimination are widespread [63]. In both experiments, anti-fat attitudes such as "fat is bad" and "thin is good" may have been primed. In Experiment 2, at the long SOA, participants may have deliberately elected to continue processing this anti-fat bias despite having sufficient time to formulate a different response. The reason for the persistence of the congruence effect at the long SOA in Experiment 2 but not in Experiment 1 remains unclear. It is acknowledged, however, that the absence of the congruence effect at the long SOA in Experiment 1 may have been an artifact of the small sample size. In both experiments, the congruence effect at the short delay is consistent with previous research providing evidence for automatic activation of attitudes toward nonbody-related words [24]. The current findings also accord with and extend previous research demonstrating automatic affective responses upon brief exposure to body-related images [38]. The converging findings for body-related words and images highlight that even brief encounters with appearance-focused stimuli are capable of activating immediate emotional responses.

### Automatic Evaluation and Body Image Concerns

Contrary to expectation, individual differences in body image concerns were not associated with the index of automatic evaluation in Experiment 1, and extreme high scores on the measure of appearance schematicity did not influence automatic affective responses in Experiment 2. The current findings are consistent with two studies which used the Implicit Association Test [50] to assess implicit attitudes toward fatness and thinness. Strong implicit negative attitudes toward fatness and positive attitudes toward thinness were evident, irrespective of individual differences in dietary restraint [64] and thin internalisation [65].

One possible explanation for the null effect for the individual differences is that the priming tasks tapped the shared societal associations between "thinness and good" and "fatness and bad" that most females have been exposed to from an early age [64]. This is consistent with the notion that most females have developed "universal" body image schemas containing rudimentary thoughts and feelings about one's physical appearance [29]. Most females, through socialisation experiences, are likely to have developed well-rehearsed, schematic associations in memory between body-related concepts and negative and positive affect. Hence, when a relevant stimulus is briefly encountered, it may be that for most women, irrespective of individual body image concerns, body-related concepts and affect are activated from memory [see [66]], such that a negative affective response is triggered in the presence of a "fat" concept and a positive affective response is activated in the presence of a "thinness" concept.

Consistent with the notion that automatic evaluation can influence higher order evaluative judgments [18], automatic evaluation of body-related words may influence subsequent, consciously-monitored appearance-related evaluative processes and behaviours. For instance, automatic evaluation may underlie the widespread tendency for females to engage in conscious negative evaluation of their bodies [67], including fat talk [68] or to pursue maladaptive behaviours such as restrictive dieting. Furthermore, automatic evaluation may be one of the mechanisms involved in the maintenance and perpetuation of anti-fat bias and discrimination. For example, one experiment has demonstrated that negative attitudes toward fatness predict subsequent interpersonal behaviour. That is, stronger implicit anti-fat attitudes were associated with individuals choosing to sit further away from an overweight individual [14]. It is also possible that higher order judgments, such as social comparison processes, may influence automatic evaluation of body-related stimuli. Conscious, intentional evaluations of body-related stimuli and behaviour were not examined in the current research. Hence, the potential bidirectional relationship between automatic processes and higher order judgements is currently speculative. Future research that parallels assessment of implicit racial attitudes [e.g., [69]], is required to determine the potential link between automatic evaluation, conscious processing and behaviour, including fat talk, fat prejudice, discrimination, and dieting.

### Limitations and Future Directions

There were several limitations of the current research. First, the two samples consisted exclusively of undergraduate females who are not representative of the general population. Second, the sample size was relatively small in both experiments, particularly in Experiment 1. Clearly the findings require replication in larger, community-based samples and in females from other age groups. Third, if body image concerns do influence automatic evaluation, it is likely to be a small effect and it is acknowledged that there was insufficient power in both experiments to detect such effects. Fourth, explicit responses toward the body words were not obtained. Hence, the potential relationship between automatic (implicit) evaluation and explicit attitudinal measures could not be examined. In addition, the memory instructions that were included in both experiments to ensure that participants attended to the primes may have inadvertently created a cognitive load. Future research could test the impact of effortful processing on automatic evaluation of body-related stimuli by having participants complete the priming task twice, with and without a competing cognitive load.

Finally, it could be argued that a "good" or "bad" judgment is not the first that automatically comes to mind when women encounter body-relevant words. For example, females may automatically process the aesthetic appeal of a body-related concept (e.g., "beautiful" versus "ugly"), prior to or even instead of the valence of the stimulus. If this is the case, then individual differences, such as thin internalisation, may be more likely to emerge as moderators of "attractiveness-oriented" automatic processing. Moreover, there is evidence that evaluations assessed by indirect measures can also be influenced by the current context and the focus of attention [70].

## Conclusions

The current research highlights that females of college age automatically evaluate body-related words under conditions of brief exposure. This study is the first to demonstrate that body shape messages delivered through the symbolic media of visual language can elicit automatic evaluation of body image stimuli, and that such effects are not reliably associated with body image concerns. These findings, together with the evidence for automatic affective processing of body-related images [38], suggest that affective responses are likely to be triggered in most young females by a variety of body-related concepts, which are often only briefly encountered. Moreover, because the majority of our encounters with media messages are fleeting, the current evidence for young females' automatic processing of these stimuli highlights their "pervasiveness (and invasiveness)" [17, p. 340].

## Competing interests

The authors declare that they have no competing interests.

## Authors' contributions

KJW conceived of and designed the experimental studies, carried out the research, performed the statistical analyses, and drafted the manuscript. JC participated in the conceptualisation and design of the studies, and helped to draft the manuscript. Both authors read and approved the final manuscript.

## Pre-publication history

The pre-publication history for this paper can be accessed here:

http://www.biomedcentral.com/1471-2458/10/308/prepub
